# A Fragmented Rib as the First Clue to Advanced Lung Adenocarcinoma

**DOI:** 10.1002/rcr2.70201

**Published:** 2025-04-29

**Authors:** Hiroshi Ishii, Takuhide Utsunomiya, Yoshiaki Kinoshita, Hisako Kushima

**Affiliations:** ^1^ Department of Respiratory Medicine Fukuoka University Chikushi Hospital Fukuoka Japan

**Keywords:** adenocarcinoma, bone metastasis, CT imaging, fragmented rib, lung cancer

## Abstract

Severe rib destruction on initial imaging can be the first clue to advanced lung adenocarcinoma with skeletal metastases, even before respiratory symptoms appear.

## Graphical abstract

Osteolytic bone metastases are common in advanced lung cancer, particularly adenocarcinoma; however, here we present a rare case of a patient presenting with dramatic rib fragmentation. This case highlights the importance of recognising uncommon radiographic features that may reflect underlying malignancy.

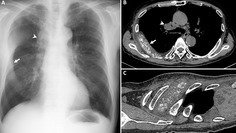

A man in his 80s with a history of chronic smoking presented with left shoulder pain. Computed tomography (CT) of the shoulder incidentally revealed nodules in the right upper lobe of the lung. Chest radiography showed a banana‐shaped opacity and a tumorous shadow in the right lung field (Figure 1A). Chest CT identified a fragmented and markedly enlarged rib, consistent with osteolytic metastasis (Figure 1B,C). Additionally, CT showed a right upper lobe mass, pulmonary nodules, and mediastinal lymphadenopathy.

**FIGURE 1:**
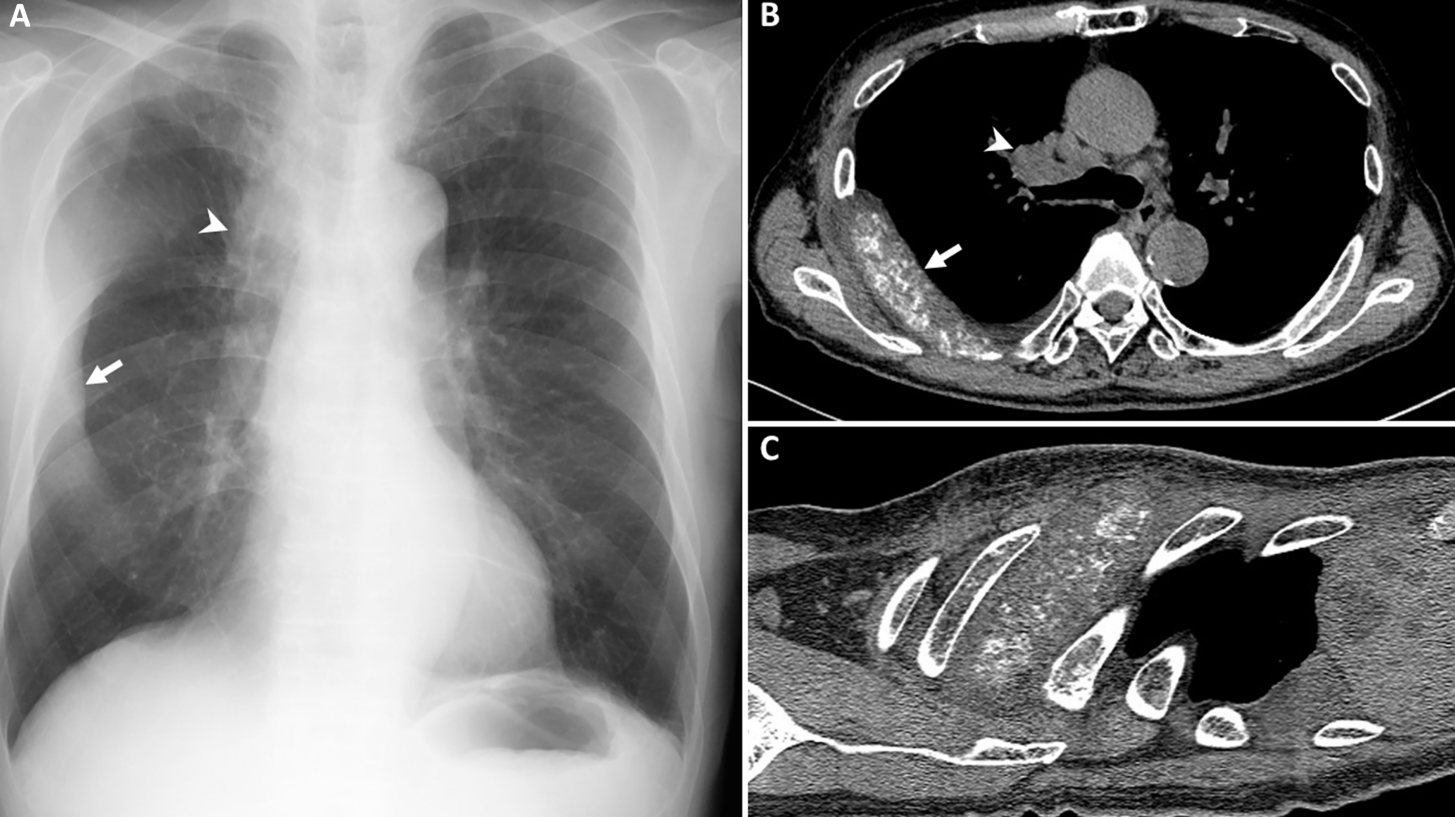
(A) Chest radiography showing a banana‐shaped opacity (arrow) and a tumorous shadow (arrowhead) in the right lung field. Axial (B) and sagittal (C) computed tomographic scans of the chest showing a mass in the upper lung lobe (arrowhead) and a fragmented and enlarged rib (arrow) due to metastasis.

Laboratory testing revealed elevated carcinoembryonic antigen (502 ng/mL; normal ≤ 5) and alkaline phosphatase (1125 U/L; normal ≤ 322), suggestive of active tumour burden and bone involvement. A biopsy confirmed primary lung adenocarcinoma. Whole‐body evaluation revealed multiple skeletal metastases to vertebrae and ribs.

Osteolytic bone metastases are common in advanced lung cancer [1, 2], particularly adenocarcinoma, but such dramatic rib fragmentation at presentation is rare. It can easily be misinterpreted or overlooked if clinicians are not vigilant. This case highlights the importance of recognising uncommon radiographic features that may reflect underlying malignancy.

## Author Contributions

**H.I.:** patient evaluation, manuscript drafting, image selection, final approval of the manuscript. **T.U.:** investigation; writing – review and editing, visualisation, final approval of the manuscript. **Y.K.:** writing   review and editing, final approval of the manuscript. **H.K.:** writing – review and editing, final approval of the manuscript.

## Consent

The authors declare that written informed consent was obtained for the publication of this manuscript and accompanying images using the consent form provided by the Journal.

## Conflicts of Interest

The authors declare no conflicts of interest.

## Data Availability

Research data are not shared.
